# Subtle Increases in Interletter Spacing Facilitate the Encoding of Words during Normal Reading

**DOI:** 10.1371/journal.pone.0047568

**Published:** 2012-10-17

**Authors:** Manuel Perea, Pablo Gomez

**Affiliations:** 1 ERI-Lectura and Departament de Metodologia, Universitat de València, Valencia, Spain; 2 Psychology Department, DePaul University, Chicago, Illinois, United States of America; University of Leicester, United Kingdom

## Abstract

**Background:**

Several recent studies have revealed that words presented with a small increase in interletter spacing are identified faster than words presented with the default interletter spacing (i.e., w a t e r faster than water). Modeling work has shown that this advantage occurs at an early encoding level. Given the implications of this finding for the ease of reading in the new digital era, here we examined whether the beneficial effect of small increases in interletter spacing can be generalized to a normal reading situation.

**Methodology:**

We conducted an experiment in which the participant’s eyes were monitored when reading sentences varying in interletter spacing: i) sentences were presented with the default (0.0) interletter spacing; ii) sentences presented with a +1.0 interletter spacing; and iii) sentences presented with a +1.5 interletter spacing.

**Principal Findings:**

Results showed shorter fixation duration times as an inverse function of interletter spacing (i.e., fixation durations were briefest with +1.5 spacing and slowest with the default spacing).

**Conclusions:**

Subtle increases in interletter spacing facilitate the encoding of the fixated word during normal reading. Thus, interletter spacing is a parameter that may affect the ease of reading, and it could be adjustable in future implementations of e-book readers.

## Introduction

The study of the perceptual factors that may facilitate the ease of reading have a long history (see [1], [2] for review; see also [3], [4] for recent evidence on the role of visual factors during web browsing and normal reading). One of these potentially relevant factors is interletter spacing. This is a feature that can be easily adjustable in current word-processing software and it applies to all characters (including spaces): compare New York vs. N e w Y o r k (default vs. +1.5 interletter spacing). A +1.5 interletter spacing refers to an expanded 1.5 inter-character spacing in MS-Word (via points to default, where a point is approximately 0.35 millimeters; see Table 1 for illustration). Importantly, three recently published studies ([5], [6], [7]), using a word identification task (“is the letter string a word?”; see [8], for a review of the lexical decision task) revealed that response times to individual words are shorter when the interletter spacing is slightly wider than the default settings (around +1.2 versus the default 0.0; e.g., w a t e r faster than water). This beneficial effect occurs not only with adult skilled readers ([5], [6], [7]), but also with young readers and with developmental dyslexics ([7], [9]).

**Table 1:** Illustration of the interletter spacing conditions in the experiment.

Interletter spacing (points to default)	Example
Default (0.0)	My cousin has bought an aquarium for her new home
Expanded (+1.0)	My cousin has bought an aquarium for her new home
Expanded (+1.5)	My cousin has bought an aquarium for her new home

Note: The original sentence in Spanish was “Mi prima ha comprado una pecera para su nueva casa”.

What is the alleged locus of the beneficial effect of small increases of interletter spacing during word processing? Perea and Gomez [6] showed, using fits from Ratcliff’s [10] diffusion model, that this effect occurs at an early encoding stage. According to their modeling work, interletter spacing affects the parameter related to stimulus encoding in the diffusion model (see [11] for the initial application of the diffusion model to the lexical decision task). Perea and Gomez [6] argued that this encoding advantage was due to two non-exclusive explanations: i) small increases in interletter spacing led to less lateral masking from the neighboring letters (i.e., less “crowding” effects), and ii) small increases in interletter spacing produced less letter position uncertainty in the letter string (see [12], [13], for two input-coding schemes which assume some position uncertainty). Unsurprisingly, when interletter spacing exceeds some critical point (e.g., as in the sequence a v o i d   t o o   m u c h   i n t e r l e t t e r   s p a c i n g), the process of word-identification – and reading speed – would be slowed down (see [14], [15]).

The goal of the present study is to examine whether subtle increases in interletter spacing also influence the eye movement pattern in normal silent reading (i.e., whether the beneficial effect of small increases in interletter spacing obtained with isolated words can be generalized to normal reading). This is important for theoretical and applied reasons. On the theoretical side, it is important to examine how small increases in interletter spacing – a factor which has been found to benefit early encoding in visual-word recognition experiments – affect eye movement control during sentence reading. On the applied side, if reading times during normal reading are faster in those sentences with small increases of interletter spacing than with the sentences with the default settings, this would suggest that current word-processing software may not employ the optimal settings. Keep in mind that the default settings in the current publishing companies and e-book applications have been set without a clear empirical basis [16], and hence it may not be optimal –note that unlike word-processing software, current e-book readers do not allow changes in the default interletter spacing settings.

Previous eye movement research on normal silent reading has generally replicated the effects previously obtained in visual-word identification experiments ([17]; see also [18], [19] among others). If a small increase in interletter spacing produces a more efficient encoding stage on the fixated word during normal silent reading because of a decrease in “visual crowding”, this may lead to shorter fixation durations. Nonetheless, the generalization to normal reading may not be straightforward as with other phenomena. Small increases in interletter spacing can also have a deleterious effect during sentence reading because the non-fixated words will be more distant from the fixation than when the interletter spacing is smaller. This may lead to a decline in acuity when processing the words in the parafovea which may lead to a larger number of (re)fixations, thus producing slower (total) reading times (i.e., “visual acuity” explanation). In this respect, neither of the two most influential models of reading – the E-Z Reader model [20] and the SWIFT model [21] – makes direct predictions because perceptual factors such as interletter spacing are beyond the scope of their current implementations.

In the present experiment, one-line sentences were presented with the default interletter settings or with small increases of interletter spacing while the participants’ eye movements were monitored. It was conducted on a CRT screen –note that using an e-reader would not have allowed for precise eye-tracking. To our knowledge, no published studies have systematically examined the effect of small increases of interletter spacing during normal silent reading while the participants’ eye movements are monitored. Nonetheless, we should note that, in a recent experiment, Paterson and Jordan [15] examined the combined effect of increases in interletter and interword spacing in normal reading. Specifically, in one of the key conditions, they added an extra space between all letters of a word in a proportional font (Courier), as in t h e T h a i r e s t a u r a n t. This added space interfered with the normal process of reading, relative to the default condition (i.e., the Thai restaurant) in average fixation durations and total reading times, among other variables. Leaving aside that the interletter spacing manipulation was rather large (around +7.0) – which could have hindered the words’ integrity – the increases to interletter spacing in that condition also decreased the saliency of interword spaces. Indeed, the reading cost relative to the default condition was smaller when an extra space as added between words (e.g., t h e   T h a i   r e s t a u r a n t). In fairness to Paterson and Jordan [15], we should indicate that the focus of their experiment was not to examine the beneficial effects of increases of interletter spacing, but rather the examination of the inter-play between inter-letter and inter-word spacing.

The participants’ eyes were monitored when reading sentences varying in interletter spacing (see Table 1 for illustration): i) sentences were presented with the default (0.0) interletter spacing; ii) sentences presented with a +1.0 interletter spacing; and iii) sentences presented with a +1.5 interletter spacing. These three values of interletter spacing were chosen on the basis of the lexical decision experiment of Perea and Gomez ([6]; see also [5], [7]). Perea and Gomez found a linear decrement of word identification times when presenting words with −0.5, 0.0, +0.5, +1.0, and +1.5 interletter spacings. As in the lexical decision experiments of Perea and cols, sentences were presented in a widely used font (14-pt Times New Roman).

To examine the role of interletter spacing during normal reading, we recorded global measures on each sentence such as reading times, average fixation durations, and number of fixations. In addition, to explore in greater detail the pattern of eye movements when interletter spacing is manipulated, local measures of a target word embedded in the sentences were also analyzed. In particular, we examined the duration of the initial fixation (first fixation duration), the duration of all fixations before leaving the target word (gaze duration), the total time (the sum of all fixations on the target word; i.e., including regressive saccades), and the initial landing position on the target word. Importantly, we also manipulated the frequency of a target word embedded in the sentence (low-frequency vs. high-frequency). Word-frequency is a well-studied predictor of the ease of word-identification in visual-word recognition and reading. If the effect of interletter spacing occurs in an early encoding process (see [6]), the effects of word-frequency and interletter spacing should not interact (see also [5], for evidence of additive effects in lexical decision).

## Results

EyeDoctor software (http://www.psych.umass.edu/eyelab/software/) was employed to process the raw eye-tracking data. All fixations shorter than 80 ms that were within one letter of the next/previous saccade were combined into that fixation, and subsequently, all fixations with individual fixations shorter than 80 ms or longer than 800 ms were excluded (less than 4%). The statistical analyses were conducted over participants (F_1_) and items (F_2_). List (list 1, list 2, list 3) was included as a dummy factor in the analyses to partial out the variability due to the counterbalancing lists [22]. The average eye movement data for the global and local analyses are displayed in Tables 2 and 3, respectively.

**Table 2:** Global measures for each of the conditions in the experiment (means and standard deviations): total sentence reading time (in ms), progressive saccade length (in number of characters), forward/backward fixation duration (in ms), and number of forward/backward fixations.

	Total reading time		Saccade length		Fixation duration				Number of fixations			
					Forward		Backward		Forward		Backward	
Interletter spacing	M	SD	M	SD	M	SD	M	SD	M	SD	M	SD
Default (0.0)	2119	406	7.6	0.8	228	22	216	29	7.4	1.1	2.1	0.8
Expanded (+1.0)	2068	398	7.5	0.8	221	20	205	21	7.4	0.9	2.1	0.8
Expanded (+1.5)	2103	408	7.3	0.9	216	20	205	31	7.7	1.0	2.2	0.9

**Table 3:** Local measures (target word) for the different experimental conditions in the experiment (mean and standard deviation): First fixation duration (in ms), Gaze duration (in ms), Total time (in ms), Initial Landing Position (range 0–5), and Skipping rate (in %).

	First fixation duration				Gaze duration				Total time				Landing position				Skipping rate			
	Low-frequency		High-frequency		Low-frequency		High-frequency		Low-frequency		High-frequency		Low-frequency		High-frequency		Low-frequency		High-frequency	
Interletter spacing	M	SD	M	SD	M	SD	M	SD	M	SD	M	SD	M	SD	M	SD	M	SD	M	SD
Default (0.0)	227	24	227	34	284	45	256	44	366	77	310	61	2.0	0.5	2.1	0.5	12.9	12.3	9.2	10.7
Expanded (+1.0)	227	25	222	30	295	35	264	40	359	69	306	50	1.8	0.4	1.7	0.6	10.1	11.4	8.3	6.7
Expanded (+1.5)	219	22	212	24	291	51	264	38	352	80	328	73	1.8	0.5	1.6	0.4	10.6	9.0	6.9	7.6

### Global Measures

#### Total reading time

The Analyses of Variance (ANOVAs) on total reading time revealed a significant effect of interletter spacing only in the participant analysis, F_1_(2, 42) = 3.85, MSE = 5679.4, η ^2^ = .16, p = .03; F_2_(2, 234) = 1.80, MSE = 28820.3, η ^2^ = .02, p = .16. Pairwise comparisons revealed that there were no signs of a difference between the default condition and the +1.5 condition, F_1_(1, 21) = 1.11, p = .30; F_2_(1, 117) <1, p = .95. More important, reading times were shorter in the +1.0 spacing condition than in the default condition in the participant analysis (2068 vs. 2119 ms, respectively), F_1_(1, 21) = 6.31, MSE = 6747.4, η ^2^ = .23, p = .02; F_2_(1, 117) = 2.32, MSE = 32003.8, η ^2^ = .02, p = .13, and the +1.5 spacing condition in the item analysis (2068 vs. 2103 ms, respectively), F_1_(1, 21) = 3.28, MSE = 5307.0, η ^2^ = .14, p = .08; F_2_(1, 117) = 3.93, MSE = 20541.5, η ^2^ = .03, p = .050.

#### Average fixation duration (progressive saccades)

The ANOVA on the average fixation durations following progressive saccades revealed a significant effect of interletter spacing, F_1_(2, 42) = 31.05, MSE = 25.8, η ^2^ = .60, p<.001; F_2_(2, 234) = 25.23, MSE = 88.7, η ^2^ = .18, p<.001: fixation durations were shorter in the +1.5 spacing condition than in the +1.0 spacing condition (216 vs. 221 ms, respectively), F_1_(1, 21) = 10.33, MSE = 25.5, η ^2^ = .33, p = .004; F_2_(1, 117) = 12.38, MSE = 72.2, η ^2^ = .10, p = .001, and in turn, fixation durations were shorter in the +1.0 spacing condition than in the default condition (221 vs. 228 ms, respectively), F_1_(1, 21) = 24.43, MSE = 22.8, η ^2^ = .54, p<.001; F2(1, 117) = 14.43, MSE = 94.7, η ^2^ = .11, p<.001.

#### Average fixation duration (regressive saccades)

The ANOVA on the fixation durations following regressive saccades also revealed a significant effect of interletter spacing, F_1_(2, 42) = 5.45, MSE = 200.8, η ^2^ = .21, p = .008; F_2_(2, 234) = 5.00, MSE = 810.2, η ^2^ = .04, p = .007. This reflected shorter fixation durations in the +1.5 condition than in the default condition (205 vs. 216 ms, respectively), F_1_(1, 21) = 9.09, MSE = 185.0, η ^2^ = .30, p = .007; F_2_(1, 117) = 5.36, MSE = 1019.7, η ^2^ = .04, p = .02, and briefer fixation durations in the +1.0 expanded condition than in the default condition (205 vs. 216 ms, respectively), F_1_(1, 21) = 8.20, MSE = 195.3, η ^2^ = .28, p = .009; F_2_(1, 117) = 10.67, MSE = 620.6, η ^2^ = .06, p = .001. There were virtually no differences in the average fixation duration of regressive saccades between the +1.5 and +1.0 conditions, both Fs<1, both ps >.79.

#### Number of (progressive) saccades

The ANOVA on the number of progressive saccades revealed an effect of interletter spacing, F_1_(2, 42) = 15.37, MSE = 0.045, η ^2^ = .42, p<.001; F_2_(2, 234) = 9.29, MSE = 0.28, η ^2^ = .07, p<.001. This reflected that there were more progressive fixations in the +1.5 condition than in the +1.0 condition (7.69 vs. 7.41, respectively), F_1_(1, 21) = 17.79, MSE = 0.052, η ^2^ = .46, p<.001; F_2_(1, 117) = 18.31, MSE = 0.19, η ^2^ = .14, p<.001, and in the default condition (7.69 vs. 7.36, respectively), F_1_(1, 21) = 38.22, MSE = 0.029, η ^2^ = .65, p<.001; F_2_(1, 117) = 12.37, MSE = 0.35, η ^2^ = .10, p = .001. The number of fixations was similar in the +1.0 and the default conditions, both Fs<1 and both ps >.67.

#### Progressive saccade length (defined as the actual number of characters)

The ANOVA on the length of progressive saccades revealed an effect of interletter spacing, F_1_(2, 42) = 14.91, MSE = 0.039, η ^2^ = .42, p<.001; F_2_(2, 234) = 5.15, MSE = 0.38, η ^2^ = .04, p = .006. (Progressive saccade length was smaller in the +1.5 condition than in the +1.0 condition (7.27 vs. 7.52, respectively), F_1_(1, 21) = 10.99, MSE = 0.062, η ^2^ = .34, p = .003; F_2_(1, 117) = 5.53, MSE = 0.47, η ^2^ = .05, p = .02, and in the default condition (7.27 vs. 7.58, respectively), F_1_(1, 21) = 39.98, MSE = 0.026, η ^2^ = .66, p<.001; F_2_(1, 117) = 15.07, MSE = 0.22, η ^2^ = .11, p<.001. The saccade length in the default condition and in the +1.0 condition did not differ significantly (7.58 vs. 7.52, respectively), F_1_(1, 21) = 1.21, p = .28; F_2_(1, 117) <1, p = .79.

#### Number of (regressive) saccades

The ANOVA on the number of regressive fixations did not reveal any significant effects, both Fs <1, both ps >.47.

### Local Measures

#### First fixation duration

The ANOVA on the first fixation durations revealed a significant effect of interletter spacing, F_1_(2, 42) = 5.83, MSE = 596.1, η ^2^ = .22, p = .006; F_2_(2, 224) = 5.54, MSE = 723.6, η ^2^ = .05, p = .004. This reflected shorter fixation durations in the +1.5 condition than in the default condition (215 vs. 227 ms, respectively), F_1_(1, 21) = 8.89, MSE = 649.9, η ^2^ = .30, p = .007; F_2_(1, 112) = 10.17, MSE = 734.5, η ^2^ = .08, p = .002, and the +1.0 expanded condition (215 vs. 225 ms), F_1_(1, 21) = 10.23, MSE = 447.6, η ^2^ = .33, p = .004; F_2_(1, 112) = 6.95, MSE = 580.0, η ^2^ = .06, p = .01. There were no differences between the default and the +1.0 conditions, both Fs <1, both ps >.30. First-fixation durations were, on average, 5 ms faster for high-frequency words than for low-frequency words, but this difference was not statistically significant, F_1_(1, 21) = 2.80, MSE = 520.9, η ^2^ = .12, p = .11; F_2_(1, 112) = 1.53, MSE = 1224.4, η ^2^ = .01, p = .21. Finally, there were no signs of an interaction between the two factors, both Fs<1, both ps >. 56.

#### Skipping rate

The ANOVA on the percentage of skipping rate revealed that high-frequency words were skipped more often than low-frequency words, F_1_(1, 21) = 6.77, MSE = 54.0, η ^2^ = .24, p = .02; F_2_(1, 112) = 6.42, MSE = 176.3, η ^2^ = .05, p = .01. (Thus, the lack of reliable effect of word-frequency in first-fixation durations was not due to some shallow processing of the target words; indeed, as indicated below, there was a robust effect of word-frequency for gaze durations.) Target words in the default condition were skipped more often (11.0%) than in +1.0 or +1.5 conditions (9.2 and 8.8%, respectively), but the main effect of inter-letter spacing was not significant, F_1_<1, p = .46; F_2_(2, 224) = 1.00, p = .36. The interaction between the two factors was not significant either, both Fs <1, both ps >.40.

#### Gaze durations

The ANOVA on the gaze durations only revealed an effect of word-frequency (261 vs. 290 ms, for high- and low-frequency words, respectively), F_1_(1, 21) = 21.52, MSE = 2681.9, η ^2^ = .51, p<.001; F_2_(1, 112) = 13.09, MSE = 5421.4, η ^2^ = .11, p<.001. Neither the effect of interletter spacing (F_1_(2, 42) = 2.13, MSE = 606.8, η ^2^ = .09, p = .13; F_2_(2, 224) <1, p>.41) nor the interaction between the two factors (both Fs <; both ps>.57) approached significance.

#### Total time

The ANOVA on the total times only revealed an effect of word-frequency (315 vs. 359 ms, for high- and low-frequency words, respectively), F_1_(1, 21) = 25.93, MSE = 5171.8, η ^2^ = .55, p<.001; F_2_(1, 112) = 10.75, MSE = 15220.0, η ^2^ = .09, p = .001. The effect of interletter spacing was not significant (both Fs <1, both ps >.50). Finally, the interaction between the two factors was significant in the analyses by items, F_1_(2, 42) = 1.97, MSE = 2084.4, η ^2^ = .19, p = .15; F_2_(2, 224) = 3.28, MSE = 4349.6, η ^2^ = .03, p = .04. This reflected some reading cost for high-frequency words in the +1.5 spacing condition (see Table 3).

#### Initial landing position

The number of letters of the target words in the experiment varied between six and nine. For that reason, before conducted the analyses of initial landing position, each word was divided into five fixation areas of the same size (see [23], [24] for similar analyses).The ANOVA on the landing position only revealed a significant effect of interletter spacing, F_1_(2, 42) = 10.20, MSE = 0.41, η ^2^ = .33, p<.001; F_2_(2, 234) = 14.17, MSE = 0.41, η ^2^ = .11, p<.001. This reflected that initial landing position on the target word in the default condition was closer to the middle of the target word than in the +1.0 condition (2.1 vs. 1.8, respectively), F_1_(1, 21) = 7.48, MSE = 0.59, η ^2^ = .26, p = .012; F_2_(1, 117) = 14.85, MSE = 0.46, η ^2^ = .12, p<.001, or the +1.5 condition (2.1 vs. 1.7, respectively), F_1_(1, 21) = 16.11, MSE = 0.47, η ^2^ = .43, p<.001; F_2_(1, 117) = 21.08, MSE = 0.50, η ^2^ = .16, p<.001. There were no differences between the +1.0 and the +1.5 conditions_(1.8 vs. 1.7, respectively) F_1_(1, 21) = 2.66, MSE = 0.15, η ^2^ = .11, p = .11; F_2_(1, 117) = 1.40, MSE = 0.26, η ^2^ = .01, p>.20. Neither the effect of frequency (F_1_(1, 21) = 1.30, MSE = 0.072, η ^2^ = .06, p = .27; F_2_<1, p = .40) nor the interaction between the frequency and spacing (F_1_(2, 42) = 1.85, MSE = 0.129, η ^2^ = .08, p = .17; F_2_<1, p = .63) approached significance.

## Discussion

The present experiment examined how small increases in interletter spacing affected eye movement control during normal silent reading relative to the default settings. We found shorter average fixation durations for the sentences with small increases in interletter spacing, both at the local level and the global level – thus providing a replication of the lexical decision experiments of Perea and cols. ([5], [6], [7]) using a more ecologically valid procedure.

Consistent with the experiments using isolated words (see [5], [6], [7]), there is an early encoding advantage on the fixated word when there is a slight increase in interletter spacing - note that 22 out of 24 participants showed an advantage of small increases in interletter spacing. At the local level, this beneficial effect of small increases in interletter spacing occurred in the first fixation durations on the target words, whereas it disappeared in gaze durations and total times. The effect of interletter spacing did not interact with word-frequency. If one applies the additive-factor logic (see [5], [15], [23], [24] for a similar reasoning), this suggests that the facilitative effect of interletter spacing on the fixated word appears to occur at an early stage of processing (see also Perea et al. [5], for a similar finding in a visual-word recognition task). This interpretation is consistent with the claim that interletter spacing affects the encoding of letters in words within the context of accumulation of evidence models that assume an encoding process that extracts information to be used in the decision making process during the process of lexical access (see [6] for modeling work on the diffusion model). We should note that, unlike the present experiment, Paterson and Jordan [15] found an interaction between interletter spacing and word-frequency in the local measures for gaze durations, with less frequent words being hindered by large increases in interletter spacing than more frequent words. However, as discussed by Perea et al. [5], the interaction occurred mainly because of the condition in which there were no obvious boundary cues between (as in t h e T h a i r e s t a u r a n t), which affected substantially more the low-frequency than the high-frequency words. In the present experiment, the manipulation of the interletter spacing occurred for all characters, including spaces (see Table 1) so that between-word boundaries were well-defined.

The data from the average fixation durations might be taken to suggest that there would be a beneficial effect of small increases in interletter spacing in total reading times, consistent with the processing advantage at encoding the fixated words. However, the story is more complex. Increases in interletter spacing were also accompanied by more fixations and shorter saccade lengths (in number of characters), especially in the +1.5 condition. This deleterious effect of interletter spacing is probably related to the fact that the initial landing position in the target word in the increased interletter spacing conditions was closer to the beginning to the word than to the optimal viewing position (i.e., the position in the word –just before the word center– in which performance is superior in word recognition tasks; see [25]). This may have led to more (re)fixations, thus cancelling the initial advantage of the small increases in interletter spacing during a very early encoding stage –note that the presence of a shorter average fixation duration when the fixation is to the left of the optimal viewing position (i.e., an inverted optimal viewing position effect) is not new (e.g., see [26]), and it reflects the interplay between lexical and oculomotor factors during normal reading. The detrimental effect of increases of interletter spacing is also consistent with the decrease in acuity of the neighboring words (e.g., parafoveal preview benefits), especially in the +1.5 interletter spacing condition, in which the saccade length was shorter than in the other conditions. Although the lack of a significant difference for the skipping data suggests that there was no effect of the spacing manipulation on parafoveal preview benefit, the skipping rate was slightly lower for the +1.5 interletter spacing condition than for the default condition (8.8 vs. 11.0%, respectively). Thus, the benefits to gaze durations and total times may not have emerged because the benefits of easier encoding (which are reflected in the average fixation durations) were outweighed by difficulties in integrating the word as a whole when the spacing was somewhat large –in particular in the +1.5 spacing condition. As an anonymous reviewer indicated, a similar dissociation has been made when examining the role of inserting spaces in normally concatenated German compounds: Inhoff, Radach, and Heller ([27]) found initial benefits of the spaced compounds (e.g., initial fixation duration) but later costs –Inhoff and cols attributed to difficulties in understanding the compound as a whole.

In the present experiment, total reading times were slightly faster when the letters within a word were slightly more separated (+1.0 interletter spacing condition) than with the default settings. Nonetheless, we are cautious in making strong inferences from the total reading data because: i) the observed effect was numerically small in magnitude; ii) only two-thirds of the participants showed this advantage in total reading times; and iii) the effect did not reach the significance level in the item-analysis –note that the effect was barely significant (p = .029) when using a linear mixed model analysis for this comparison; and iv) the effect did not occur for total reading times or gaze durations at the local (word) level. Clearly, a question for further research is the role of the individual differences in the choice of the optimal interletter spacing during normal reading using paragraphs (rather than single-text sentences) and a psychophysical approach. In order to increase the applicability of the present findings, we applied the interletter manipulation to all characters (i.e., as current word processors do). Nonetheless, it may be of interest to examine experimentally how subtle increases in interletter spacing interact with interword spacing, thus extending the work of Paterson and Jordan [15].

What are the implications of the present findings? At the theoretical level, the present experiment has revealed that eye movement control is sensitive to a rather subtle manipulation of interletter spacing. This was clearly so on the decision of when to move the eyes (i.e., average fixation durations): small increases in interletter spacing appear to facilitate the encoding the target word. This is consistent with the “visual crowding” account stated in the Introduction –i.e., interletter spacing would affect the earliest stages of letter processing in the EZ Reader or SWIFT models. Importantly, the fact that the effect of interletter spacing did not appear in gaze durations at the local level does suggest that some effort was required in the conditions with an increased interletter spacing which cancelled out the initial encoding benefit. Indeed, the interletter spacing manipulation affected the decision of where to move the eyes: the initial landing position was closer to the beginning of the word for the words with increases interletter spacing. As an anonymous reviewer suggested, landing position differences could be the result of readers failing to accommodate their oculomotor behavior to account for the increased distance between words in the conditions with increased interletter spacing –in particular in the +1.5 interletter spacing condition in which the saccade length was smaller than in the other two conditions.

At the practical level, the present data suggest that the choice of the default interletter spacing made by publishing/software companies may not be the optimal settings. Further research should be conducted to examine the potential benefits of small increases of interletter spacing across a variety of devices not just with adult skilled readers, but also in other populations. In a recent study, Perea et al. [7] found a clear advantage of interletter spacing with a group of individuals with developmental dyslexia, both in the lexical decision times and in the total reading times of a continuous reading task. (The eye movements were not monitored in that study, though.) Importantly, when the parallel experiments were conducted with a group of control children, the benefit of interletter spacing only occurred in the lexical decision times, but not in the total reading times of the continuous reading task ([7]; see also [9] for similar evidence in Italian and French). This suggest that the potential benefits of interletter spacing in the early stages of processing for readers with dyslexia overcome the potential disadvantage of increasing interletter spacing at the parafoveal level – probably because the deleterious effects of crowding are typically greater for readers with dyslexia than for an age-control group (e.g., see [28]). Additional caution is required if one wants to generalize the obtained findings to many of the devices used for reading e-books (e.g., Kindle, iPad). These devices have some differences (besides portability) with the CRT screen typically used in eye-tracking experiments like the present one: the resolution is better, which leads to less anti-aliasing, which might alter fixation patterns (see [4]). In addition, we only employed one font, Times New Roman (i.e., the same employed in our previous experiments on this issue, see [5], [6], [7]). Different fonts may have a different interletter spacing by default, and Times New Roman is a more narrowly spaced font (e.g., compare finding vs. finding; Times New Roman vs. Calibri). Clearly, the choice of font may qualify the optimal interletter settings – note that fonts may also differ in a number of other aspects (e.g., x-height, absence/presence of serifs, etc; see [4] for an analysis of the effects of fonts during normal reading).

In sum, the present experiment has revealed that eye movement control in adult skilled readers is affected by subtle increases in interletter spacing. In particular, fixation durations on the fixated word are shorter when there is a small increase in interletter spacing. This suggests that interletter spacing is a relevant parameter when processing text, and implementations of e-book applications should include an option to modify interletter spacing –as is currently the case in most word-processing applications. More research should be committed to examine in greater depth how interletter spacing affects eye movement control during normal reading with other populations (e.g., young children learning to read, readers with dyslexia, see [7], [9]).

## Materials and Methods

### Ethics Statement

All participants gave informed consent in writing – the experiment was conducted with the approval of the “Comité Ético de Investigación en Humanos de la Comisión de Ética en Investigación Experimental de la Universitat de València” (Ethics Committee for Human Research at the University of Valencia).

### Participants

Twenty-four students (average age: 20 years) from the University of Valencia participated in the experiment. They received a small monetary compensation (3 €). They were native speakers of Spanish, had normal (or corrected-to-normal) vision, and were naïve as to the purpose of the experiment.

### Apparatus

The participants’ eye movements were recorded using an Eyelink II eyetracker (SR Research Ltd, Canada). This is a video-based eye tracking device with cameras that sample pupil location at a 500-Hz rate. Viewing was binocular, but only the data from the right eye were registered. The average gaze position error is less than 0.5°. The sentences were presented on a 22-inch ViewSonic Professional series P225f CRT monitor. The participant’s situation with respect to the monitor was controlled by a head-tracking camera that served for compensating possible head motion. Participants were seated 70 cm from the screen. At this distance, the number of letters in 14-pt Times New Roman font in 1° of visual angle was approximately 3.71 for the default spacing, 3.47 for the +1.0 interletter spacing, and 3.05 for the +1.5 interletter spacing –note that Times New Roman is not a monospaced font so that different letters may correspond to different angles.

### Materials and Design

We employed the 120 sentences created by Perea and Acha [24]. For each sentence, there was a target word which could be of low-frequency (average frequency: 4.5 per million in the B-Pal database [29]) or of high-frequency (average frequency: 87.3 per million) of the same length, as in “Mi prima ha comprado una pecera/alarma para su nueva casa” and “El vendedor ha colocado una pecera/alarma en el escaparate”; “pecera” [fishbowl] is a low-frequency word, and alarma [alarm] is a high-frequency word). The average number of letters of the target words was 7.3 (range: 6–9). Two counterbalanced sets of stimuli were created so that participants read the 120 target words in one of the two sentence frames (e.g., for a given participant, the sentence “Mi prima ha comprado una pecera para su nueva casa” [the low-frequency word pecera is the target word], would be accompanied by the sentence “El vendedor ha colocado una alarma en el escaparate”, in which high-frequency word would appear in the other sentence frame). All sentences were easily comprehensible in Spanish (as deduced from the results of a norming task), and all target words had a low predictability (less than 5% in a “cloze” task; see [24] for additional details). The list of sentences is available at [24]. For each participant, forty sentences were presented with the default interletter spacing (i.e., 0.0 interletter spacing; twenty with a low-frequency target word, and twenty with a high-frequency target word), forty sentences with a +1.0 interletter spacing (twenty with a low-frequency target word, and twenty with a high-frequency target word), and forty sentences with a +1.5 interletter spacing (twenty with a low-frequency target word, and twenty with a high-frequency target word) (see Table 1). Interletter spacing was counterbalanced across three lists for each sentence frame. The order of the sentences was randomized for each participant.

### Statistical Analyses

Two types of analyses were carried out. For the three interletter spacing conditions (0.0, +1.0, +1.5), we examined six global measures: reading time (in ms), average fixation duration [for both progressive and regressive saccades], saccade length (in number of characters), and number of saccades [for both progressive and regressive saccades]). For the local measures on the target word, we examined the first fixation duration (i.e., the duration of the initial fixation on the target word), skipping rate (i.e., the percentage of skipping the target word), the gaze duration (i.e., the sum of all fixations on the target word before leaving it), the total time (i.e., the sum of all fixations on the target word, including regressive saccades), and the initial landing position as a function of interletter spacing (0.0, +1.0, +1.5) and word-frequency (low, high).

### Procedure

Each participant sat in front of the computer monitor in a quiet, dimly lit room. Participants were instructed to read the sentences silently for comprehension, as they would normally do. They were also asked to press a button in a game-pad when they finished reading each sentence. Before proceeding with the experiment, each participant was instructed to follow several dots on the computer monitor to calibrate the eye tracker. Then, each sentence was displayed on a single line of text. Eight practice trials were employed to familiarize the participants with the procedure, and this was followed by the 120 experimental sentences. Before the presentation of each sentence, the eye tracker was checked –it was recalibrated when necessary. The sequence in each trial was the following. A black square appeared on the left-hand side of the monitor. Once the participant looked at that square, the sentence appeared. The location of the square corresponded with the initial letter of the sentence. To ascertain that participants were actually reading the sentences for comprehension, yes/no questions were asked after 20% of the sentences. Participants had no difficulty responding to these questions (error rates were less than 4% and similarly distributed across conditions). Sentences were presented in a different random order for each participant.
